# Magnetic Iron–Improved Growth, Leaf Chemical Content, Yield, and Fruit Quality of Chinese Mandarin Trees Grown under Soil Salinity Stress

**DOI:** 10.3390/plants11212839

**Published:** 2022-10-25

**Authors:** Khadiga Alharbi, Khalid S. Alshallash, Ashraf E. Hamdy, Sobhy M. Khalifa, Hosny F. Abdel-Aziz, Ahmed Sharaf, Walid F. Abobatta

**Affiliations:** 1 Department of Biology, College of Science, Princess Nourah Bint Abdulrahman University, Riyadh 11671, Saudi Arabia; 2 College of Science and Humanities-Huraymila, Imam Mohammed Bin Saud Islamic University (IM SIU), Riyadh 11432, Saudi Arabia; 3 Department of Horticulture, Faculty of Agriculture, Al-Azhar University, Cairo 11884, Egypt; 4 Soils and Water Department, Faculty of Agriculture, Al-Azhar University, Cairo 11884, Egypt; 5 Citrus Department, Horticulture Research Institute, Agriculture Research Center, Giza 12619, Egypt

**Keywords:** (*Citrus reticulata* blanco), salt stress, fruit quality, yield increasing, vitamin C

## Abstract

Chinese mandarin fruits are an inexpensive and rich source of vitamin C. They have potential benefits in treating acute respiratory infections and mitigating inflammation in critical patients with COVID-19. In Egypt, citrus is the most important fruit tree but is sensitive to salinity stress, resulting in poor vegetative tree growth and reductions in productivity and fruit quality. Magnetic iron has emerged as a promising approach in the citrus tree industry, since it improves vegetative growth, yield, and fruit quality and alleviates salinity stress in Chinese mandarin trees grown in soils suffering from high salt stress. This research is aimed at studying the influence of adding magnetic iron (as soil treatment) on tree canopy growth, yield, and fruit quality of ‘Chinese’ mandarin trees. Therefore, the treatments were as follows: 0, 250, 500, and or 750 g of magnetic iron.tree^−1^. Our results indicated that all applications of magnetic iron significantly improved tree canopy volume, leaf total chlorophyll, relative water content, yield (kg.tree^−1^), and the fruit physical and chemical characteristics of Chinese mandarin. In contrast, leaf Na and Cl content, (%), proline, and total phenolic content were decreased by magnetic iron soil treatments. In respect to vegetative growth, our results indicated that adding magnetic iron at the concentration 750 g.tree^−1^ caused the best values of tree canopy volume. A similar trend was noticed regarding yield. The increase in yield attained was nearly 19%; the best values were obtained when magnetic iron were used at 750 g.tree^−1^. In conclusion, the application of magnetic iron can lead to improved fruit production and fruit quality of Chinese mandarin trees grown in salinity stress conditions.

## 1. Introduction

Mandarin fruit has a high nutritional value [1]. This fruit contains ascorbic acid (vitamin C), which improves immunity and general health in humans [2]. Such a vitamin represents one of the most crucial micronutrients of mankind [3]. Ascorbic acid possesses potential benefits in treating acute respiratory infections and mitigating inflammation in many critical diseases, such as COVID-19 [4]. Chinese mandarin (*Citrus reticulata* Blanco) is widely cultivated among citrus fruit trees worldwide. Usually, the fruits are eaten fresh; thus, the internal quality of fruit, such as peel color, fruit weight, juice volume, and easy peeling, play an important role in marketing the fruit [5]. Citrus cultivators thus seek increased yield and improved fruit quality [6].

Although citrus is a most important cash fruit crop and is grown under semi-arid conditions [7], it has vulnerability to salinity stress [8]. Such stress leads to poor vegetative tree growth and reductions in productivity and fruit quality [9].

Magnetic iron (magnetite) is a natural row rock that has very high iron content, black color, and a hardness rating 6 on the Mohs hardness scale. Magnetic iron contains 48.8% Fe_3_O_4_, 17.3% FeO, 26.7% Fe_2_O_3_, 2.6% MgO, 4.3% SiO_2_, and 0.3% CaO, [10]. The addition of natural magnetic iron improves soil structure, organic matter, water properties, and cation exchange capacity [11]. Such iron enhances soil energetic and vigorous properties (magneto biology) [12], which help in plant growth, moderation of soil temperature, water holding capacity, and crop nutrition [13]. In addition, the magnetic instrument isolates all chlorine and unsafe gases from the soil [14], which expands salt development and reduces nutrient ability [15]. The application of iron as a fertilizer improves plant growth and reduces the damaging effects of environmental stresses [16].

For instance, magnetic iron treatments improved growth parameters and productivity of pepper plants [17]. Moreover, El-Desouky et al. [18] stated that magnetic iron addition at rates ranging from 0 to 100 mg.kg^−1^ soil to tomato plants significantly increased plant growth, leaf nutrients, and photosynthetic efficiency. EL Ghayaty et al. [19] reported that soil application with magnetic iron at concentrations rates (200 and 250 g/vine) increased the parameters of vegetative growth, vine yield, and fruit quality of superior grapevines. Similarly Soliman et al., [15] studied Florida prince peach trees treated with 1000 g of magnetic iron. They saw increased growth in tree^−1^, photosynthetic pigments, nutrients in leaves, yield, and physical properties of fruits, whereas leaf proline contents and fruit total acidity were decreased. In addition, magnetic iron enhanced vegetative growth and yield of olive trees under new reclaimed soil conditions [20]. 

However, limited studies are available regarding the soil application of Chinese mandarin trees with magnetic iron. Hence, this study aims to clarify the impacts of applying magnetic iron at different concentrations on canopy growth, leaf nutrient content, total chlorophyll, and fruit yield, in addition the physical and chemical properties of Chinese mandarin cultivars planted under soil salinity stress.

## 2. Results and Discussion

### 2.1. Effect of Magnetic Iron on Tree Canopy Volume

**Figure 1:**
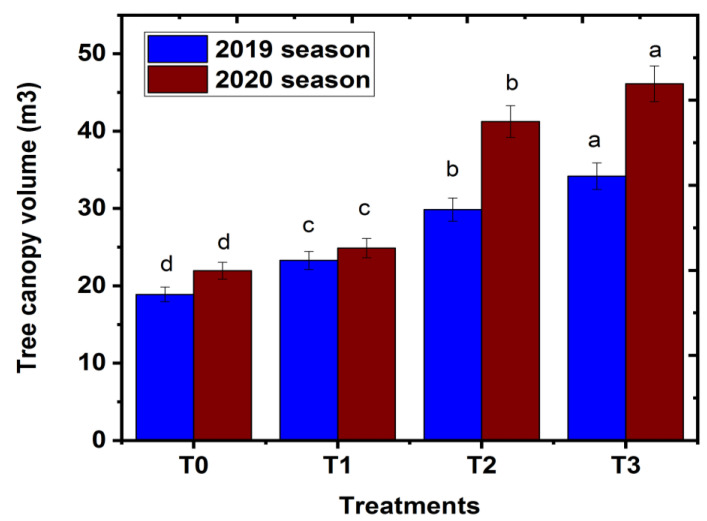
Effect of magnetic iron application on tree canopy volume (cm^3^) of ‘Chinese’ mandarin trees during 2019 and 2020 seasons. T0: 0 g magnetic iron.tree^−1^, T1: 250 g magnetic iron.tree^−1^, T2: 500 g magnetic iron.tree^−1^**,** and T3: 750 magnetic iron.tree^−1^. Bars indicate mean values ± SE (*n* = 9). Different letters above columns indicate significant differences among magnetic iron treatments at *p* = 0.05 according to Bartlett’s test.

The effect of magnetic iron soil application on the canopy volume of trees is shown in Figure 1. The results show a clear response of ‘Chinese’ mandarin trees to the application of magnetic iron in the orchard. The data in Figure 1 show that each of the magnetic iron applications significantly improved the volume of canopy in the ‘Chinese’ mandarin cultivar in both studied seasons compared with that of untreated plants. Comparing the effect of the three magnetic concentrations, the results showed a definite trend regarding tree canopy volume parameter in response to magnetic concentrations. Tree canopy under 750 g magnetic tree^−1^ was greater than that under other magnetic treatments or the control one. The increase in canopy growth might be due to the positive effect of magnetic iron on the development of several groups of microorganisms, which may excrete a range of vitamins, substances of growth, and antibiotics, which may also raise tree growth [10]. Abou-Baker et al. [21] reported that magnetic iron application produced more nutrients out of the soil, and at the same time, the oxygen level was improved by 10%, resulting in a better assimilation of nutrients and fertilizer in plants during the vegetative period. The current result is confirmed by the previous study of Abobatta, [22] who found that the application of magnetic iron at high concentrations gave maximum growth parameters of the Valencia orange cultivar. These findings are in agreement with those of EL Ghayaty et al. [19], who found that soil treatment with magnetic iron improved the vegetative growth characteristics of Superior grapevine compared with control plants.

### 2.2. Effect of Magnetic Iron on Leaf Chemical Content

**Figure 2:**
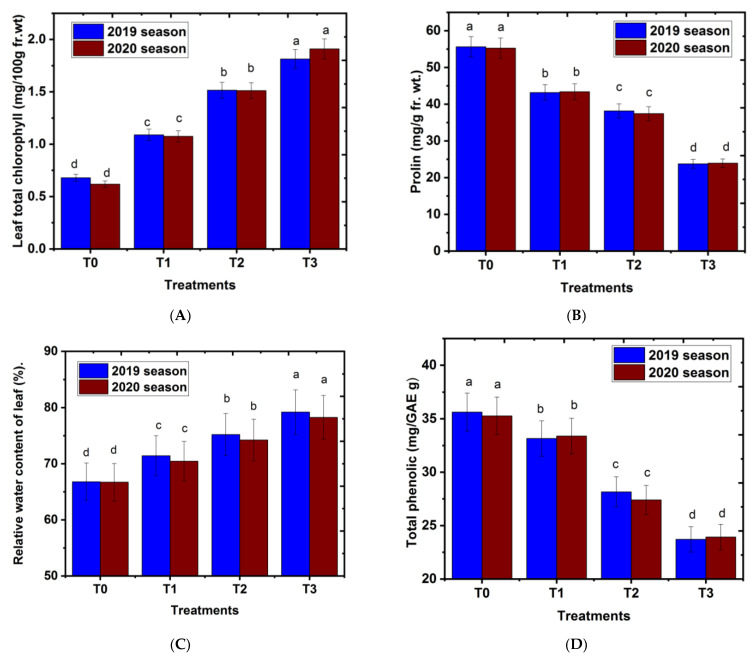
Effect of magnetic iron application on (**A**) leaf total chlorophyll content (mg.100 g^−1^ Fr.Wt), (**B**) leaf proline content (mg.100 g^−1^ Fr.Wt), (**C**) Relative water content of leaf (%). and (**D**) total phenolic (mg.GAE^−1^.g^−1^) of ‘Chinese’ mandarin trees during 2019 and 2020 seasons. T0: 0 g magnetic iron.tree^−1^, T1: 250 g magnetic iron.tree^−1^, T2: 500 g magnetic iron.tree^−1^, and T3: 750 magnetic iron.tree^−1^. Bars indicate mean values ± SE (*n* = 9). Different letters above columns indicate significant differences among magnetic iron treatments at *p* = 0.05 according to Bartlett’s test.

The results in Figure 2A–D represent the effect of magnetic iron as a soil application on the leaf total chlorophyll content, proline content, relative water content, and total phenolic content of ‘Chinese’ mandarin trees. Magnetic iron soil application significantly increased leaf total chlorophyll and relative water content (Figure 2A,C). In contrast, proline and total phenolic content were decreased by magnetic iron soil treatments of ‘Chinese’ mandarin trees compared to the control group (Figure 2B,D). Moreover, there was a defined trend that could be drawn for all leaf chemical properties: total chlorophyll and relative water content were increased with an increasing magnetic iron level, while proline and total phenolic content were decreased with increasing concentration of magnetic iron. The treatment of 750 g of magnetic iron.tree^−1^ had the best values of leaf chemical properties compared to untreated plants or other applications. It could be concluded that soil application with magnetic iron enhanced leaf chemical properties, which led to plants improving a resistant mechanism to abiotic stress, such as drought in semi-arid conditions. One of the most prevalent amino acids in citrus tissues is proline. It is one of four amino acids where accumulation peaks during stress and serves as a significant soluble nitrogen store in citrus leaves. It also plays an adaptive role as an osmotic substance [23]. These results are in agreement with Abobatta [24], who found that the leaf chemical properties of Valencia Orange were enhanced with application of magnetic iron at a concentration of 500 or 1000 g tree^−1^. Similarly, EL Ghayaty et al. [19], reported on Superior seedless grapevines at levels of 200 or 250 g magnetic iron.vine^−1^. The increase in total chlorophyll and relative water content of leaf could be attributed to the positive impact of application with magnetic iron, which plays a vital role in cation uptake capacity and has a positive effect on immobile plant nutrient uptake and increases the chemical properties of plants [25,26].

### 2.3. Effect of Magnetic Iron on Leaf Nutrient Contents

**Figure 3:**
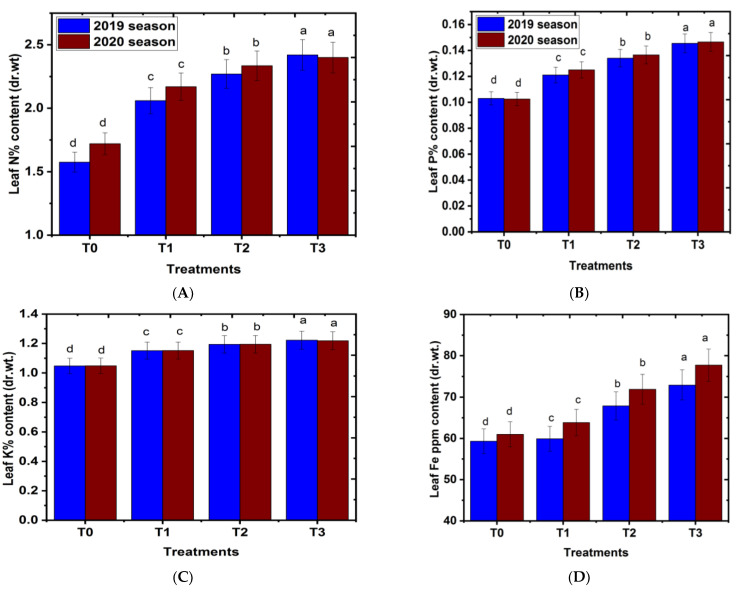

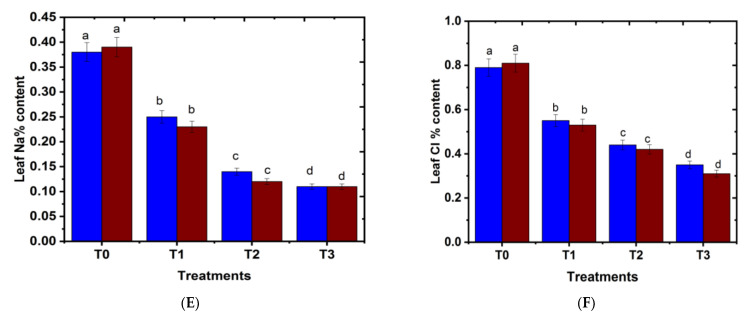
Effect of magnetic iron application on leaf nutrient content of ‘Chinese’ mandarin trees during 2019 and 2020 seasons. (**A**) Leaf N% content (dr.wt.), (**B**) leaf P% content (dr.wt.), (**C**) leaf K% content (dr.wt.), (**D**) leaf Fe ppm content (dr.wt.), (**E**) leaf Na content (%) and (**F**) leaf Cl content (%). T0: 0 g magnetic iron.tree^−1^, T1: 250 g magnetic iron.tree^−1^, T2: 500 g magnetic iron.tree^−1^, and T3: 750 magnetic iron.tree^−1^. Bars indicate mean values ± SE (*n* = 9). Different letters above columns indicate significant differences among magnetic iron treatments at *p* = 0.05 according to Bartlett’s test.

Data in Figure 3 show that magnetic iron treatment significantly increased leaf nitrogen, phosphorus, potassium, and iron (Fe) nutrient content of the Chinese mandarin when compared to the control in the two studied seasons. These findings matched those of Abobatta [24], who discovered that using magnetic iron at concentrations of 1000 g.tree^−1^ enhanced leaf mineral content in Valencia orange leaves compared to the control. In a similar line, Soliman et al. [15] discovered that applying magnetite at a concentration of 1000 g.tree^−1^ once significantly enhanced leaf N, P, and K contents in Florida prince peach trees as compared to the control. Trees given magnetic iron at a concentration of 750 g.tree^−1^ yielded the highest significant values of nitrogen, phosphorus, potassium, and iron, while the control treatment yielded the lowest value in both seasons. These results are in agreement with Ahmed [27], who found that magnetic iron application increased leaf N, P and K contents of grape cv. Superior compared with the control. The improvement in these parameters could be attributed to the effect of magnetic iron, which included a change in the physiochemical characteristics of soil, resulting in improved dissolvability of different chemical elements, more salts out of the soil, and a 10% increase in oxygen concentration, resulting in better nutrient and fertilizer assimilation in plants during the vegetation period. It also resulted in greater nutrient and fertilizer uptake in plants during the vegetative phase, as well as increased crop output [28,29]. Furthermore, the results showed that magnetic iron application significantly increased both leaf Na and Cl content (%) compared to the untreated group (Figure 3E,F). The reduction in leaf Na and Cl contents due to the use of magnetic iron improved the soil’s ability to eliminate salts [30]. It could be concluded that magnetic iron application resulted in a decrease in both leaf Na and Cl content, which are considered the most important elements harmful to the trees, leading to the protection of trees from the harmful effect of salinity and improved growth and fruiting. Furthermore, the magnetic process removes all chlorine and poisonous and dangerous gases from the soil and increases salt flow and nutrient solubility, which aids plant growth and moderates soil temperature [31]. In many cases, enough Fe is translocated from the roots to the leaves, and the efficiency of the leaf Fe is critical [32]. There is now enough evidence that this efficiency is related to the pH in the leaf apoplast and to the activity of the plasma membrane–located Fe reductase [33]. See also ref. [34].

### 2.4. Effect of Magnetic Iron on Yield kg.Tree^−1^

**Figure 4:**
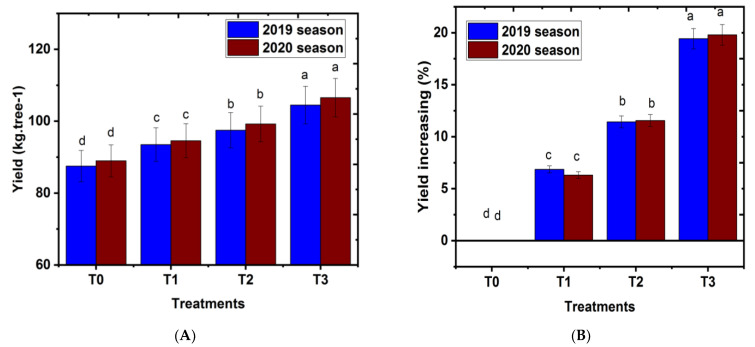
Effect of magnetic iron application on (**A**) yield kg.tree^−1^ and (**B**) yield increasing (%) of ‘Chinese’ mandarin trees during 2019 and 2020 seasons. T0: 0 g magnetic iron.tree^−1^, T1: 250 g magnetic iron.tree^−1^, T2: 500 g magnetic iron.tree^−1^, and T3: 750 magnetic iron.tree^−1^. Bars indicate mean values ± SE (*n* = 9). Different letters above columns indicate significant differences among magnetic iron treatments at *p* = 0.05 according to Bartlett’s test.

The results in Figure 4 clearly show that all applied magnetic iron treatments to mandarin tree cv. Chinese caused a significant increase in fruit yield (kg.tree^−1^) when compared with untreated trees (control) in both seasons. The best results regarding magnetic iron application in mandarin trees were gained when magnetic (750 g.tree^−1^) followed descending order by 500 g.tree^−1^ and 250 g.tree^−1^. On the other hand, the lowest fruit yield (kg.tree^−1^) was recorded when mandarin trees received treatment (control). A similar trend was observed regarding yield increase (%), which was superior to untreated trees in both seasons. The increase in yield attained was nearly 19% compared with the control. These results are in agreement with those gained by Abobatta [24], who reported that magnetic treatments at concentrations of 500 and 1000 g.tree^−1^ significantly increased the yield of ‘Valencia’ cv. orange under Egyptian environmental conditions. Similarly, Abd El-Rhman [35] found that magnetic iron treatments in the growth season increased yield/trees in the Manfalouty pomegranate cultivar. Moreover, Mohamed [36] found that magnetic treatments at concentrations of 250 g.tree^−1^ increased yield in ‘Keitt’ cv. mango in comparison to that of the control. The increase in fruit yield/tree might be due to the rise in weight of fruit, fruit volume, and number of fruits retained/tree at harvest as compared with those of the control [37]. Magnetic iron affected numerous parameters of the plants, such as root growth, shoot growth, reproduction, and development of the meristem cells and total chlorophyll content, which in turn increased yield due to the enhancing effect on the anabolic processes occurring in the plant, which improves the quality of fruits [38]. Moreover, the increments in yield/tree by magnetic iron application might be due to the vital roles in tree growth resulting from magnetic iron application, such as improving soil structure, water properties, holding capacity of water, and capacity of cation exchange, as well as the increased nutrition from nutrients. Furthermore, nutrient solubility in water caused an increase in plant growth by the magnetic process [31,39]. Magnetic iron application significantly increased yield and improved element uptake in the root area profile [17]. The increase in yield might be due to the increase in retained fruit percentage, improved nutritional status, and subsequent appropriate vegetative growth of the treated navel orange trees [40]. Moreover, magnetic iron plays a beneficial role in the assimilation and absorption of nutrients, which raises fruit quality and yield [29].

### 2.5. Effect of Magnetic Iron on Some Fruit Physical Properties

#### 2.5.1. Fruit Weight and Volume

**Figure 5:**
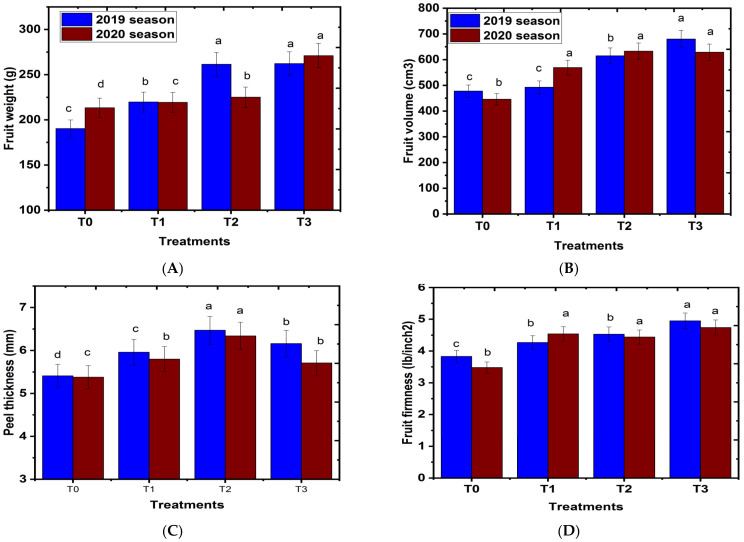

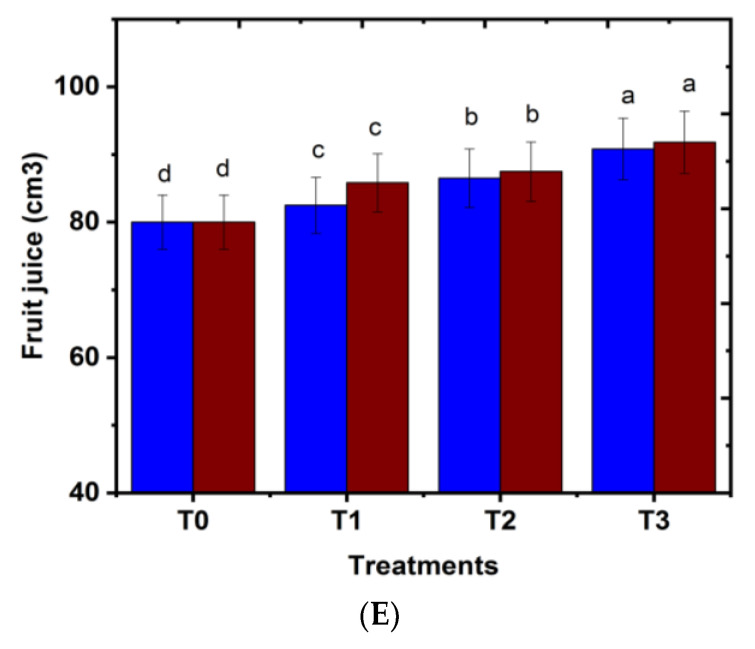
Effect of magnetic iron application on fruit physical properties of ‘Chinese’ mandarin trees during 2019 and 2020 seasons: (**A**) fruit weight, (**B**) fruit volume, (**C**) peel thickness, (**D**) fruit firmness(lb/inch^2^), and (**E**) fruit juice. T0: 0 g magnetic iron.tree^−1^, T1: 250 g magnetic iron.tree^−1^, T2: 500 g magnetic iron.tree^−1^, and T3: 750 magnetic iron.tree^−1^. Bars indicate mean values ± SE (*n* = 9). Different letters above columns indicate significant differences among magnetic iron treatments at *p* = 0.05 according to Bartlett’s test.

Magnetic iron treatment significantly increased fruit physical parameters such as fruit weight, fruit volume, and fruit peel thickness of the ‘Chinese’ mandarin variety when compared with that of the control in both seasons (Figure 5A,B). In most cases, treatment with 750 g of magnetic iron.tree^−1^ was superior to other treatments or the control regarding the above-mentioned characteristics. The stimulatory impact of magnetic iron application on fruit weight and fruit volume reported in this study is in agreement with that obtained by Hoda et al. [41], who found that application of magnetic iron significantly improved the average fruit weight and volume of ‘Manfalouty’ pomegranate trees in comparison with those of the control. The same applied to Hamdy et al. [37] on Balady and Fremont mandarin trees. Mohamed [36] also found that magnetic treatments at concentrations of 250 g.tree^−1^ increased fruit weight in ‘Keitt’ cv. mango in comparison to that of control. The positive impact of adding magnetic iron as a soil application on fruit weight could be because the magnetic field plays an important role in cation uptake capacity and has a positive effect on immobile plant nutrient uptake [29]. Additionally, the magnetic process removes all poisonous and dangerous gases, including chlorine, from the soil, increases the movement of salt and the solubility of nutrients, increases soil water retention, which aids in plant growth, and moderates soil temperature [31]. Magnetic iron application on plants significantly enhanced vegetative growth characteristics [18]. This could be a resonance-like phenomenon, which increases the inner energy of the plant and may help fruit weight to increase [42,43].

#### 2.5.2. Fruit Firmness

The influence of tested applications on fruit firmness of ‘Chinese’ mandarin are presented in Figure 5D. The results show that magnetic iron significantly caused an increase in firmness of ‘Chinese’ mandarin fruits compared with untreated trees. The best results regarding magnetic iron application in mandarin trees were gained when magnetic iron was applied at 750 g.tree^−1^, followed in descending order by 500 g.tree^−1^. These results are in harmony with those obtained by Soliman et al. [15], who reported that magnetic iron treatment increased fruit firmness of peach trees when compared with that of the control. Magnetic iron has been reported to have a positive effect on plant growth and development [44,45]. The increase in some physical parameters of ‘Chinese’ mandarin cultivar might be due to the positive effect that magnetic iron plays in immobile plant nutrient uptake, cation uptake capacity, and yield production [25]. Magnetic iron compound treatments increased fruit size, which may be attributed to the fact that magnetic iron contains silicon that causes an increase in the absorption of potassium, which maintains the plant water status [10].

#### 2.5.3. Fruit Juice Volume

Data presented in Figure 5E showed that magnetic iron significantly increased averages of fruit juice volume of ‘Chinese’ mandarin fruits as compared to that of untreated trees in both seasons. Magnetic iron at a concentration of 750 g.tree^−1^ achieved the highest values in comparison to those of other treatments. The stimulatory result of magnetic iron on fruit juice volume reported in this study is incompatible with El-Dengawy et al. [40], who stated that magnetic iron treatments significantly increased fruit weight and volume of ‘Washington’ navel orange fruits when compared with that of untreated trees. The same applies to Ennab [6] and Mohamed et al. [36] for mandarin trees. The positive effect of magnetic iron application on fruit juice might be because plants treated with magnetic iron absorb irrigation water more than non-treated plants, therefore improving nutrient uptake by plant organs, which plays a significant role in enhancing fruit quality properties [29]. It could be concluded that magnetic iron treatments applied to ‘Chinese’ mandarin trees in mid-January caused an improvement in some fruit physical properties in comparison to that of the control.

### 2.6. Effect of Magnetic Iron on Some Fruit Chemical Properties

#### 2.6.1. Total Soluble Solids (T.S.S.%)

**Figure 6:**
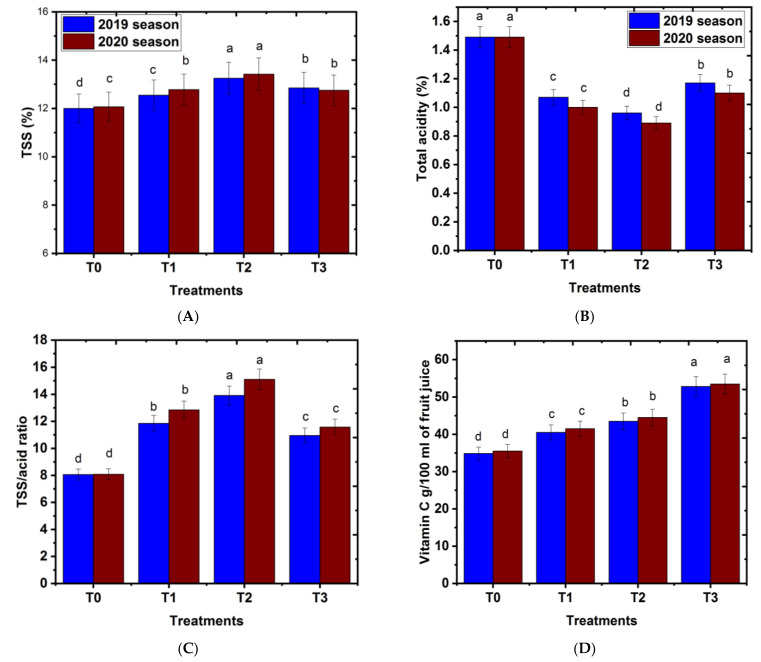
Effect of magnetic iron application on fruit chemical properties of ‘Chinese’ mandarin trees during 2019 and 2020 seasons. (**A**) TSS%, (**B**) total acidity, (**C**) TSS/acid ratio, and (**D**) vitamin C. T0: 0 g magnetic iron.tree^−1^, T1: 250 g magnetic iron.tree^−1^, T2: 500 g magnetic iron.tree^−1^, and T3: 750 magnetic iron.tree^−1^. Bars indicate mean values ± SE (*n* = 9). Different letters above columns indicate significant differences among magnetic iron treatments at *p* = 0.05 according to Bartlett’s test.

Data in Figure 6 shows that the TSS percentage of fruits significantly increased when magnetic iron was used in the ‘Chinese’ mandarin variety in comparison with the control. These results are compatible with the earlier work of Mohamed et al. [36], who stated that the TSS (%) of Balady mandarin fruit juice increased by magnetic iron treatments at concentrations of 500 and 750 g.tree^−1^ compared to that of untreated trees. Similarly, application of magnetic irrigation water increased the TSS of Hayany date palm trees [46]. Mohamed [41] found that magnetic treatments at concentrations of 250 g.tree^−1^ increased fruit TSS parentage in ‘Keitt’ cv. mango in comparison to that of untreated trees. Likewise, El-Dengawy et al. [40] stated that magnetic iron treatments significantly increased the TSS% of ‘Washington’ navel orange fruits when compared to the untreated trees. The increase in TSS percentage might be due to increasing ion mobility and ion uptake of magnetic iron treatments, which also leads to better photosynthesis stimulation in plants and an improvement of fruit characteristics [15]. Moreover, magnetic iron can change water properties, and significantly increase leaf total chlorophyll [47].

#### 2.6.2. Total Acidity% and TSS/Acid Ratio

Total acidity (%) of fruits was significantly decreased by the addition of magnetic iron treatments in both study seasons in comparison with that of the control. However, the TSS/Acid ratio of fruits increased under magnetic iron treatment in the ‘Chinese’ mandarin variety in comparison with the control (Figure 6A,C). The best results regarding magnetic iron application in mandarin trees were gained when magnetic iron was (500 g.tree^−1^). These results are in line with the findings of Mohamed et al. [41], who stated that the TSS/acid ratio of Balady mandarin fruit juice increased by magnetic iron treatments at concentrations of 500 and 750 g.tree^−1^ compared to that of the control. Similarly, El-Dengawy et al. [40] stated that magnetic iron treatments significantly increased the TSS/acid ratio of ‘Washington’ navel orange fruits. However, the total acidity (%) of fruits decreased in comparison with that of the control; see Figure 6B. This increase in TSS/Acid ratio may be due to the increase in ion mobility and ion uptake, which was previously improved under magnetic iron and in turn leads to a better photosynthesis rates in plants [47].

#### 2.6.3. Vitamin C

Vitamin C, known as Ascorbic acid/100 mL, in fruit juice significantly increased under magnetic iron treatment in the “Chinese” mandarin variety in comparison with that of the control (Figure 6D). The best results regarding magnetic iron application in mandarin trees were gained when magnetic iron was (750 g.tree^−1^), followed in descending order by 500 g.tree^−1^ and 250 g.tree^−1^. On the other hand, the lowest percentage of vitamin C in fruits was recorded when mandarin trees received no treatment (control). These results are supported with the previous study of El-Dengawy et al. [40], who found that magnetic iron treatments significantly increased vitamin C in ‘Washington’ navel orange fruits. The increase in vitamin C (Ascorbic acid mg/100 mL of fruit juice) in fruit juice at harvest time might be due to the increase in weight of the fruit and the increase in the parameters of vegetative growth, such as shoot length and leaf area, caused by magnetic iron treatment; it also may be due to irrigation with magnetized water, causing a significant increase in the activities of the antioxidant enzymes over the control plants [48].

It could be concluded that applying magnetic iron treatments to ‘Chinese’ mandarin trees mid-January improved some fruit chemical properties in comparison to that of the control.

### 2.7. Principal Components Analysis (PCA)

**Figure 7:**
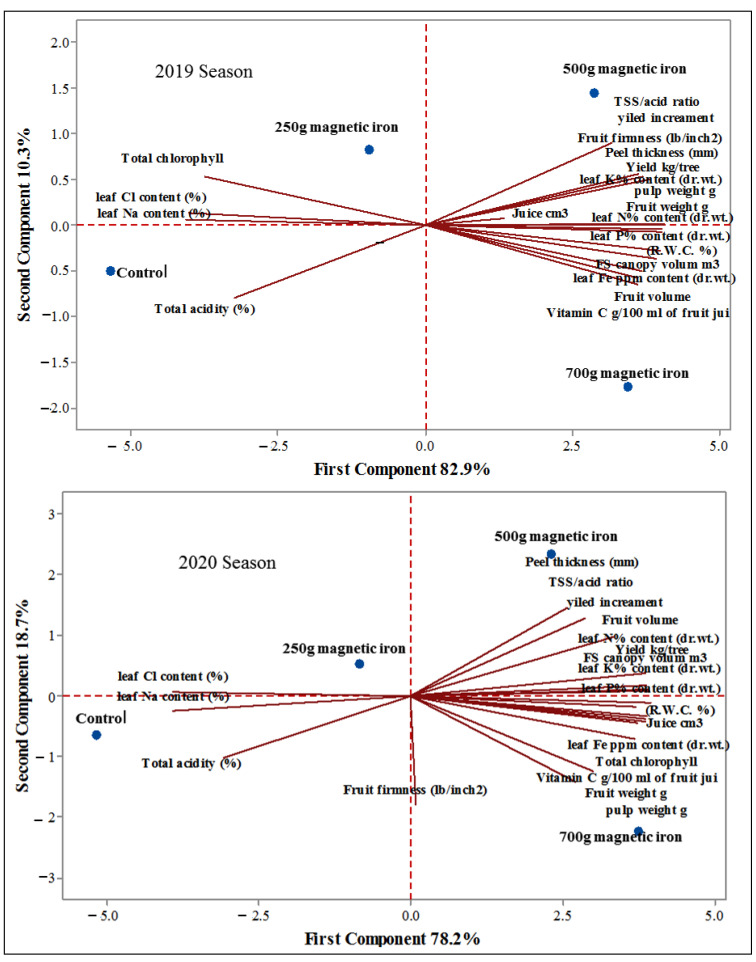
Biplot extracted by principal component analysis (PCA) among parameters studied based on different amounts of effect of magnetic iron application in 2019 and 2020 seasons: control, 250 g of magnetic iron tree^−1^, 500 g of magnetic iron tree^−1^, and 700 g of magnetic iron tree^−1^: Yield kg.tree^−1^, yield increment; Fruit weight, g; Fruit volume, fruit firmness (lb/inch^2^); pulp weight, g; juice, cm^3^; canopy volume, m^3^; leaf N% content, dr.wt.; leaf P% content, dr.wt.; leaf K% content, dr.wt.; leaf Fe ppm content, dr.wt.; leaf Na content, %; leaf Cl content, %; total chlorophyll, R.W.C.%; total acidity, %; TSS/acid ratio, Vitamin C g/100 mL of fruit juice; and peel thickness, mm.

PCA clarified the first two components of 20 variables, where the first and second components were (82.9–10.3%) and (78.2–18.7%), with a total variance of (93.2%) and (96.8%) during 2019 and 2020 seasons as a score plot; see Figure 7 and Table 1. Explants of magnetic iron application are mostly located at the upper right side of the plot and have a strong positive correlation with the first component: yield kg.tree^−1^, yield increment, fruit weight (g), fruit volume, fruit firmness (lb/in^2^), pulp weight (g), Juice cm^3^, canopy volume (m^3^), leaf N% content (dr.wt.), leaf P% content (dr.wt.), leaf K% content (dr.wt.), leaf Fe ppm content (dr.wt.), (R.W.C.%), TSS/acid ratio, vitamin C (g/100 mL of fruit juice), and peel thickness (mm) at the level of 500 g of magnetic iron tree^−1^ and 700 g of magnetic iron tree^−1^ are located at the center of the plot, while other characteristics with the level of 250 g of magnetic iron tree^−1^ are mostly located at the left side of the plot.

Correlation analysis was conducted among the measured parameters of the effect of magnetic iron application on fruit physicochemical properties of ‘Chinese’ mandarin trees during 2019 and 2020 seasons (Table 2 and Table 3). Correlation analysis of the measured parameters meant a highly significant relationship between them.

## 3. Materials and Methods

### 3.1. Experimental Site

**Figure 8:**
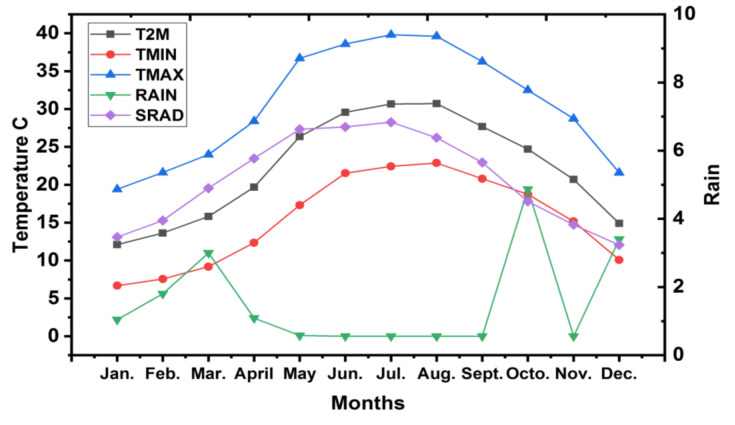
The average monthly temperature. (T2M): Temperature Average at 2 m (°C); (TMIN): Temperature at 2 m Minimum (°C); (TMAX) Temperature at 2 m Maximum (°C); (RAIN) Precipitation (mm); and (SRAD) Solar Radiation (MJ/m^2^/day) during the growing season.

This study was carried out during two successive seasons of 2019 and 2020 in the private orchard located at Wady Elmoullk, Ismailia, Governorate, Egypt (30° 26′ 16.8″N, 31° 46′ 37.92″ E). The average monthly precipitation and temperature from 2019 to 2020 according to the Central Laboratory for Agricultural Climate are presented in Figure 8. The type of the soil was classified as sandy, as shown in Table 4.

### 3.2. Experimental Design

This work was conducted from January 2019 to December 2020. The experiments were assigned to four treatments that comprised four different levels (0, 250, 500 and 750 g.tree^−1^) of magnetic iron. Each magnetic iron treatment consisted of three replications (9 trees. treatment^−1^) and was designed in accordance with the randomized block experimental design. The magnetic iron was added to the soil under irrigation lines at 20 cm depth in both sides of trees in mid-January in each season for one time only.

The Chinese mandarin cultivar (*Citrus reticulata* Blanco) was planted in 2013 after grafting onto Volkamer lemon (*Citrus volkameriana* Ten. and Pasq.) rootstock. The trees were planted 2 × 4 m apart (525 trees/fed), and surface methods of drip irrigation were used in the research farm, with 8 adjustable discharge emitters/tree (8 litter/h) through 2 irrigation lines. The distance of the irrigation line in relation to the trunk of the plant was 50 cm for each side. The region’s climate is Mediterranean, with an annual average temperature of 21.3 °C and an annual rainfall of 26 mm. The soil of the studied area is sandy (94.72% sand).

The ‘Chinese’ mandarin trees also received the recommended fertilization program (1000 g N, 1500 g P_2_O_5_, and 500 g K_2_O g.tree^−1^ year^−1^); micronutrients were applied in a mixture of 300, 150, 100, 50 and 50 mg of the applied fertilizer from chelated Fe, Mn, Zn, Cu, and B as boric acid, respectively, in March, May, and August. The recommended fertilization program was applied to all trees on an equal basis according to the extension of the Ministry of Agriculture in Egypt.

### 3.3. Field and Laboratory Measurements

#### 3.3.1. Vegetative Growth

The tree canopy volume (m^3^) was measured as one of the parameters of the vegetative growth of the plant in order to estimate the response of the growth of Chinese trees to the magnetic iron treatments. Therefore, tree canopy volume (m^3^) size, known as a canopy volume, was calculated using the formula of Zekri [49], as follows: 0.52 × tree height × (diameter^2^).

#### 3.3.2. Leaf Chemical Contents

##### Total Chlorophyll

Leaf content of total chlorophyll was taken in September, measured by using a nondestructive Minolta chlorophyll meter SPAD 502 for the apical 5th leaf according to Wood et al. [50]. In brief, the SPAD is a machine with two shafts, flexible and rigid. The flexible shaft holds the leaves onto the rigid shaft. The flexible shaft includes two diodes emitting two light beams through the leaf tissues; red light (at 650 nm) and near-infrared light (at 940 nm). The rigid shaft contains two detectors detecting the light transmittance. Based on the green tone in the leaves, the transmitted light is transformed into electric signals. The transmission ratio in the two wavelength regions is converted into a numeric value termed the SPAD reading. Therefore, the SPAD reading is associated with the leaf chlorophyll content.

##### Leaf Proline Content

The amino acid proline is the one that is most prevalent in citrus leaves [51]. Thus, proline (µ mole. g^−1^) fresh weight was determined in approximately 0.5 g of leaf samples from each group and was homogenized in 3% (*w*/*v*) sulphosalycylic acid; the homogenate was filtered through filter paper according to Bates et al. [52]. After adding acid ninhydrin and glacial acetic acid, the resulting mixture was heated at 100 °C for 1 h in a water bath. The reaction was then stopped with an ice bath. The mixture was extracted with toluene, and the absorbance of the fraction with the 4 mL toluene aspired from the liquid phase was read at 546 nm. The proline concentration was determined from a standard curve and calculated on a fresh weight basis (µ mol proline (g FW-1) according to Claussen et al. [53].

##### Relative Water Content

Leaf relative water content (RWC) was calculated according to the method by Claussen et al. [53]. Ten leaves were randomly chosen from the middle parts of the shoot. At first, leaves were separated from the stems, and their fresh masses (FW) were calculated. In order to measure the saturation mass (TM), they were placed into the distilled water in closed containers for 5 h under a room temperature of 22 °C, for the purpose of achieving their greatest amount of saturation mass; then, they were weighed. The leaves were placed inside an electrical oven for 48 h at 65 °C to determine dry weight (DW), and the dry weight of the leaves (DW) was obtained. All of the measurements were done by scales with 0.001 g accuracy, and the following equation was applied:

(1) $$\mathrm{RWC}\;\left(\%\right)=\frac{\mathrm{Fresh}\;\mathrm{weight}-\mathrm{Dry}\;\mathrm{weight}}{\mathrm{Saturation}\;\mathrm{weight}-\mathrm{Dry}\;\mathrm{weight}\;}\times 100$$

##### Total Phenolic Content

Ten grams of mature leaves was homogenized with 60 mL of solvent (80% aqueous ethanol, containing 1% conc. HCl). Phenolic compounds were extracted as described by Casquete et al. [54]. Extraction was performed using a magnetic mixer for 1 h in the absence of light at room temperature (25 °C) and filtered. This process was repeated twice. Excess ethanol was removed by heating at 37 °C in a rotary evaporator under vacuum. The resultant aqueous extracts (crude extracts) were combined to a final known volume, and total phenolic content (TPC) was measured spectrophotometrically (λ 760 nm) in a UV-2401PC spectrophotometer (Shimadzu Scientific Instruments, Columbia, MD, USA) using the Folin–Ciocalteu colorimetric method to determine the total phenolic content of ‘Chinese’ mandarin tree leaves according to Singleton et al. [55], expressed as (mg/g) using gallic acid as a standard.

##### Nutrient Contents of Leaves

Ten mature leaves/tree (the fifth distal leaf on the labeled shoot) were collected in September of both seasons in order to determine the following nutrient contents. Nitrogen (N) content was determined using the microkjeldahl method according to Adams [56] (T.N% dr.wt.) in leaves and roots. Phosphorus (P) was determined colorimetrically according to the method described by Murphy and Riley [57] in leaves. Potassium (K) was determined in samples by a flame photometer using the method of Thomas and Saunders [58]. Iron (Fe), Sodium(Na), and chlorine were determined using atomic absorption according to Cheng and Bray [59]. N, P, and K concentrations were expressed as percentages of sample dry matter.

### 3.4. Tree Yield

Harvesting was achieved during the regular commercial harvesting time in December of both seasons according to Ennab [6], and yield (kg.tree^−1^) was recorded. Yield increased in comparison with the untreated sample. Percentage was calculated using the equation of El-Naby et al. [60].

(2) $$Yield\;increasing\;\left(\%\right)\;=\frac{Yield\;\left(treatment\right)-yield\;\left(control\right)}{yield\;\left(control\right)}\times 100$$

### 3.5. Physical Characteristics of Fruits

At harvest, samples of ten fruits of each tree were replicated three times and then transferred to the chemical analytical laboratory of the Department of Horticulture, Faculty of Agriculture at Al-Azhar University to determine the following parameters: fruit weight (g), fruit volume (cm^3^), fruit peel weight (g), and juice volume (mm).

Fruit pulp firmness (lb./inch^2^) was Measured Using Pressure Tester (Digital Force-Gouge Model IGV-O.SA to FGV-100A, Shimpo Instruments)

### 3.6. Fruit Chemical Characteristics

#### Fruit Total Soluble Solids (TSS Percentage) and Total Fruit Acidity Percentage

Fruit juice TSS% was measured using a digital refractometer. Total acidity was determined by titration and referred to as citric acid according to A.O.A.C. [61]. Total soluble solids/acid ratio was calculated from the values of total soluble solids divided by values of total acids. Ascorbic acid (vitamin C) expressed as (ascorbic acid mg/100 mL juice) was estimated by titrating a juice sample with 2,6 dichlorophenol indophenol dye according to A.O.A.C. [61].

### 3.7. Statistical Analysis

All data obtained during both seasons were obtained using one-way ANOVA according to Snedecor and Cochran [62] and Co-stat software according to Stern (1991).

## 4. Conclusions

In conclusion, it is apparent that adding magnetic iron at concentrations of 250, 500, and or 750 g.tree^−1^ to the soil once in mid-January caused an improvement in canopy growth, total chlorophyll, relative water content, N,P,K, and Fe leaf contents, fruit yield, and fruit quality of the ‘Chinese’ mandarin cultivar. Moreover, Na and Cl, total phenolic leaf content, and total proline were reduced. Thus, magnetic iron treatment is a promising agent to improve growth, yield of fruit, and fruit physical and chemical properties of the ‘Chinese’ mandarin variety under arid and semi-arid conditions.

## Figures and Tables

**Table 1:** Results of principal component analysis (PCA) of effect of magnetic iron application on fruit chemical properties of ‘Chinese’ mandarin trees during 2019 and 2020 seasons.

Variable	PC1	PC2	PC3	PC1	PC2	PC3
Yield kg.tree^−1^	0.218	0.27	0.208	0.249	0.028	0.21
Yield increasing (%)	0.218	0.27	0.208	0.213	0.261	0.235
Fruit weight (g)	0.244	0.001	0.101	0.192	−0.332	0.127
Fruit volume (cm^3^)	0.222	−0.243	0.215	0.247	0.099	−0.134
Fruit firmness (lb/in^2^)	0.223	0.255	0.17	0.005	−0.482	−0.458
Pulp weight (g)	0.245	0.007	0.02	0.173	−0.378	−0.038
Juice (cm^3^)	0.081	0.038	−0.809	0.246	−0.103	−0.147
Canopy volume (m^3)^	0.237	−0.176	0.07	0.237	−0.05	0.425
Leaf N% content (dr.wt.)	0.242	−0.038	−0.129	0.249	0.049	−0.199
Leaf P% content (dr.wt.)	0.24	−0.024	−0.139	0.252	−0.03	−0.079
Leaf K% content (dr.wt.)	0.242	−0.024	−0.139	0.251	0.023	−0.16
Leaf Fe ppm content (dr.wt.)	0.217	−0.276	0.215	0.238	−0.119	0.308
Leaf Na content (%)	−0.245	0.03	0.009	−0.251	−0.065	0.039
Leaf Cl content (%)	−0.242	0.062	0.12	−0.251	0.021	0.144
Total chlorophyll	−0.226	0.256	0.127	0.249	−0.086	0.092
R.W.C. (%)	0.237	−0.181	−0.032	0.246	−0.11	0.109
Total acidity (%)	−0.196	−0.39	0.197	−0.198	−0.274	0.416
TSS/acid ratio	0.192	0.434	−0.059	0.183	0.342	−0.256
Vitamin C g/100 mL	0.218	−0.314	−0.093	0.236	−0.188	−0.001
Peel thickness (mm)	0.23	0.241	0.044	0.165	0.391	0.072

**Table 2:** Correlation analysis of the measured parameters of the effect of magnetic iron application on the fruit physic chemical properties of ‘Chinese’ mandarin trees during 2019.

	Yield kg.tree^−1^	Yield Increasing	Fruit Weight (g)	Fruit Volume (cm^3^)	Fruit Firmness (lb/inch2)	Pulp Weight (g)	Juice (cm3)	Canopy Volume (m3)	Leaf N% Content (dr.wt.)	Leaf P% Content (dr.wt.)	Leaf K% Content (dr.wt.)	Leaf Fe ppm Content (dr.wt.)	Leaf Na Content (%)	Leaf Cl Content (%)	Total Chlorophyll	R.W.C. (%)	Total Acidity (%)	TSS/Acid Ratio	Vitamin C	Peel Thickness (mm)
Yield kg.tree^−1^	1																			
Yield increasing (%)	1.000 **	1																		
Fruit weight (g)	0.912 **	0.912 **	1																	
Fruit volume	0.729 **	0.729 **	0.926 **	1																
Fruit firmness (lb/inch^2^)	0.999 **	0.999 **	0.926 **	0.744 **	1															
Pulp weight (g)	0.899 **	0.899 **	0.995 **	0.905 **	0.916 **	1														
Juice (cm^3^)	0.087 *	0.088 *	0.218 *	0.045 **	0.127 *	0.310 *	1													
Canopy volume (m^3^)	0.780 **	0.780 **	0.967 **	0.979 **	0.800 **	0.963 **	0.229 *	1												
Leaf N% content (dr.wt.)	0.820 **	0.820 **	0.963 **	0.874 **	0.845 **	0.983 **	0.464 *	0.954 **	1											
Leaf P% content (dr.wt.)	0.787 **	0.787 **	0.967 **	0.937 **	0.811 **	0.977 **	0.367 *	0.988 **	0.987 **	1										
Leaf K% content (dr.wt.)	0.823 **	0.823 **	0.959 **	0.861 **	0.848 **	0.981 **	0.480 *	0.946 **	1.000 **	0.982 **	1									
Leaf Fe ppm content (dr.wt.)	0.693 **	0.693 **	0.906 **	0.999 **	0.709 **	0.885 **	0.036 *	0.972 **	0.858 **	0.927 **	0.843 **	1								
Leaf Na content (%)	−0.870 **	−0.870 **	−0.991 **	−0.914 **	−0.889 **	−0.998 **	−0.338 *	−0.973 **	−0.990 **	−0.988 **	−0.988 **	−0.897 **	1							
Leaf Cl content (%)	−0.809 **	−0.809 **	−0.963 **	−0.887 **	−0.834 **	−0.982 **	−0.454 *	−0.962 **	−0.999 **	−0.992 **	−0.998 **	−0.872 **	0.990 **	1						
Total chlorophyll	−0.639 **	−0.639 **	−0.894 **	−0.921 **	−0.670 **	−0.911 **	−0.423 *	−0.966 **	−0.950 **	−0.978 **	−0.942 **	−0.920 **	0.935 **	0.959 **	1					
R.W.C. (%)	0.748 **	0.748 **	0.953 **	0.953 **	0.773 **	0.961 **	0.341 *	0.993 **	0.973 **	0.997 **	0.966 **	0.946 **	−0.975 **	−0.980 **	−0.987 **	1				
Total acidity (%)	−0.870 **	−0.870 **	−0.765 **	−0.467 *	−0.881 **	−0.797 **	−0.510 **	−0.608 **	−0.789 **	−0.690 **	−0.804 **	−0.425 *	0.775 **	0.768 **	0.561 **	−0.632 **	1			
TSS/acid ratio	0.918 **	0.918 **	0.767 **	0.470 *	0.921 **	0.784 **	0.357 *	0.589 **	0.745 **	0.653 **	0.759 **	0.426 *	−0.753 **	−0.724 **	−0.498 **	0.593 **	−0.985 **	1		
Vitamin C	0.587 **	0.587 **	0.867 **	0.931 **	0.618 **	0.879 **	0.371 *	0.959 **	0.916 **	0.959 **	0.907 **	0.935 **	−0.906 **	−0.929 **	−0.995 **	0.976 **	−0.479 *	0.418 *	1	
Peel thickness (mm)	0.980 **	0.980 **	0.937 **	0.738 **	0.988 **	0.941 **	0.281 *	0.820 **	0.898 **	0.852 **	0.903 **	0.704 **	−0.921 **	−0.886 **	−0.726 **	0.812 **	−0.928 **	0.942 **	0.669 **	1

Correlation is significant at the 5% level **; Correlation is significant at the 1% level *.

**Table 3:** Correlation analysis of the measured parameters of the effect of magnetic iron application on fruit physicochemical properties of ‘Chinese’ mandarin trees during 2020.

	Yield kg.tree^−1^	Yield Increasing	Fruit Weight g	Fruit Volume (cm^3^)	Fruit Firmness (lb/inch^2^)	Pulp Weight (g)	Juice (cm^3^)	Canopy Volume (m^3^)	Leaf N% Content (dr.wt.)	Leaf P% Content (dr.wt.)	Leaf K% Content (dr.wt.)	Leaf Fe ppm Content (dr.wt.)	Leaf Na Content (%)	Leaf Cl Content (%)	Total Chlorophyll	R.W.C. (%)	Total Acidity (%)	TSS/Acid Ratio	Vitamin C	Peel Thickness (mm)
Yield kg.tree^−1^	1																			
Yield increasing (%)	0.888 **	1																		
Fruit weight (g)	0.731 **	0.336 *	1																	
Fruit volume	0.953 **	0.899 **	0.608 **	1																
Fruit firmness (lb/inch^2^)	−0.097 **	−0.526 **	0.571 **	−0.124 *	1															
Pulp weight (g)	0.627 **	0.200 *	0.984 **	0.528 **	0.701 **	1														
Juice (cm^3^)	0.927 **	0.697 **	0.855 **	0.924 **	0.243 *	0.812 **	1													
Canopy volume (m^3^)	0.973 **	0.804 **	0.807 **	0.859 **	−0.022 *	0.698 **	0.890 **	1												
Leaf N% content (dr.wt.)	0.947 **	0.850 **	0.666 **	0.995 **	−0.022 *	0.601 **	0.954 **	0.858 **	1											
Leaf P% content (dr.wt.)	0.969 **	0.812 **	0.773 **	0.974 **	0.069 *	0.706 **	0.984 **	0.915 **	0.987 **	1										
Leaf K% content (dr.wt.)	0.957 **	0.834 **	0.712 **	0.989 **	0.019 *	0.647 **	0.970 **	0.881 **	0.998 **	0.995 **	1									
Leaf Fe ppm content (dr.wt.)	0.956 **	0.724 **	0.888 **	0.849 **	0.136 *	0.802 **	0.933 **	0.987 **	0.864 **	0.931 **	0.893 **	1								
Leaf Na content (%)	−0.977 **	−0.894 **	−0.669 **	−0.995 **	0.091 *	−0.585 **	−0.943 **	−0.906 **	−0.992 **	−0.987 **	−0.992 **	−0.897 **	1							
Leaf Cl content (%)	−0.956 **	−0.795 **	−0.768 **	−0.973 **	−0.094 *	−0.709 **	−0.987 **	−0.894 **	−0.989 **	−0.999 **	−0.996 **	−0.916 **	0.983 **	1						
Total chlorophyll	0.971 **	0.758 **	0.862 **	0.920 **	0.142 *	0.790 **	0.981 **	0.961 **	0.938 **	0.981 **	0.958 **	0.982 **	−0.952 **	−0.974 **	1					
R.W.C. (%)	0.962 **	0.730 **	0.884 **	0.900 **	0.179 *	0.816 **	0.979 **	0.961 **	0.922 **	0.972 **	0.945 **	0.987 **	−0.936 **	−0.965 **	0.999 **	1				
Total acidity (%)	−0.741 **	−0.863 **	−0.221 *	−0.899 **	0.358 *	−0.156 *	−0.694 **	−0.569 **	−0.873 **	−0.783 **	−0.839 **	−0.533 **	0.852 **	0.793 **	−0.656 **	−0.620 **	1			
TSS/acid ratio	0.711 **	0.904 **	0.103 *	0.852 **	−0.529 **	0.014 *	0.594 **	0.543 **	0.807 **	0.711 **	0.770 **	0.478 *	−0.805 **	−0.713 **	0.584 **	0.545 **	−0.981 **	1		
Vitamin C	0.898 **	0.602 **	0.941 **	0.840 **	0.351 *	0.901 **	0.979 **	0.907 **	0.879 **	0.941 **	0.907 **	0.961 **	−0.878 **	−0.939 **	0.977 **	0.984 **	−0.536 **	0.432 *	1	
Peel thickness (mm)	0.692 **	0.942 **	0.017 *	0.775 **	−0.714 **	−0.109 *	0.478 *	0.558 **	0.709 **	0.623 **	0.673 **	0.456 *	−0.741 **	−0.611 **	0.520 **	0.481 **	−0.890 **	0.959 **	0.334 *	1

Correlation is significant at the 5% level**; Correlation is significant at the 1% level *.

**Table 4:** Physical and chemical properties of the experimental farm soil.

Soil Physical Analysis					Soil Chemical Analysis		
Sand (%) ± SD		Silt (%) ± SD	Clay (%) ± SD	Soiltexture	EC (ds/m) ± SD	pH ± SD	
93.53 ± 1.115		4.22 ± 0.06083	2.25 ± 0.17474	Sand	4.30 ± 0.20817	8.15 ± 0.14189	
**Soil Chemical Analysis**							
Cations (meq/L)				Anions (meq/L)			
Ca^++^ ± SD	Mg^++^ ± SD	Na^+^ ± SD	K^+^ ± SD	So4^=^ ± SD	CI^−^ ± SD	HCo3^−^ ± SD	Co3^=^ ± SD
12.50 ± 0.955	9.50 ± 0.4817	18.85 ± 0.5437	1.12 ± 0.05508	15.47 ± 0.4087	24.50 ± 0.96043	2.00 ± 0.61011	0.00 ± 00
**Available nutrients Macro and micro elements (mg.kg^−1^)**							
N ± SD		P ± SD	K ± SD	Cu ± SD	Fe ± SD	Mn ± SD	Zn ± SD
179.0 ± 9.53939		8.48 ± 0.61011	94.00 ± 1.0	0.06 ± 0.01	1.07 ± 0.02	0.34 ± 0.05508	0.16 ± 0.96043

## Data Availability

Not applicable.
